# COMBI-EU: Real-World Evidence on Adverse Event Management and Time on Therapy with Adjuvant Dabrafenib Plus Trametinib in Patients with BRAF V600-Mutant Melanoma

**DOI:** 10.3390/cancers18040667

**Published:** 2026-02-18

**Authors:** Michael Weichenthal, Dirk Debus, Lisa Zimmer, Imke von Wasielewski, Friedegund Meier, Thomas Tüting, Markus V. Heppt, Jessica C. Hassel, Fabian Ziller, Peter Mohr, Pia Dücker, Anca Sindrilaru, Edgar Dippel, Lucie Heinzerling, Marc Bender, Manar Aoun, Magdalena Walecki, Rudolf Herbst, Yenny Angela, Rudolf Stadler, Sebastian Haferkampf, Claus-Detlev Klemke, Kjell Matthias Kaune, Johannes Wohlrab, Ulrike Leiter, Nessr Abu Rached, Jochen Utikal, Gaston Schley, Jens Ulrich, Erwin Schultz, Christoffer Gebhardt, Patrick Terheyden, Ralf Gutzmer, Dirk Schadendorf

**Affiliations:** 1 Skin Cancer Center Kiel, University Hospital Schleswig-Holstein, 24105 Kiel, Germany; 2 Department of Dermatology, Paracelsus Medical University, Nuremberg General Hospital, 90419 Nuremberg, Germany; dirk.debus@klinikum-nuernberg.de (D.D.); erwin.schultz@klinikum-nuernberg.de (E.S.); 3 Department of Dermatology, Venereology and Allergology, University Hospital Essen, 45147 Essen, Germany; lisa.zimmer@uk-essen.de (L.Z.); dirk.schadendorf@uk-essen.de (D.S.); 4 Department of Dermatology and Allergy, Skin Cancer Center Hannover, 30625 Hannover, Germany; vonwasielewski.imke@mh-hannover.de; 5 Department of Dermatology, Faculty of Medicine, University Hospital Carl Gustav Carus, Technische Universität Dresden, 01307 Dresden, Germany; friedegund.meier@uniklinikum-dresden.de; 6 Skin Cancer Center, National Center for Tumor Diseases and University Cancer Centre Dresden, 01307 Dresden, Germany; 7 Department of Dermatology, University Medical Center, 39120 Magdeburg, Germany; thomas.tueting@med.ovgu.de; 8 Department of Dermatology, Uniklinikum Erlangen, Friedrich-Alexander University Erlangen-Nürnberg (FAU), 91054 Erlangen, Germany; markus.heppt@uk-erlangen.de (M.V.H.); lucie.heinzerling@med.uni-muenchen.de (L.H.); 9 Comprehensive Cancer Center, Erlangen-European Metropolitan Area of Nuremberg (CC ER-EMN), 91054 Erlangen, Germany; 10 Bavarian Cancer Research Center (BZKF), 91052 Erlangen, Germany; 11 Department of Dermatology and National Center for Tumor Diseases (NCT), Medical Faculty Heidelberg, Heidelberg University, NCT Heidelberg, a Partnership Between DKFZ and University Hospital Heidelberg, 69120 Heidelberg, Germany; jessica.hassel@med.uni-heidelberg.de; 12 Department of Dermatology, DRK Hospital Chemnitz-Rabenstein, 09117 Chemnitz, Germany; ziller.fabian@drk-khs.de; 13 Department of Dermatology, Elbekliniken Buxtehude, 21614 Buxtehude, Germany; peter.mohr@elbekliniken.de (P.M.); marc.bender@elbekliniken.de (M.B.); 14 Department of Dermatology, Skin Cancer Center, Hospital Dortmund, 44137 Dortmund, Germany; pia.duecker@klinikumdo.de; 15 Department of Dermatology and Allergology, Skin Cancer Center, University Hospital Ulm, 89070 Ulm, Germany; mihaela-anca.sindrilaru@uniklinik-ulm.de; 16 Department of Dermatology, Ludwigshafen Medical Center, 67063 Ludwigshafen, Germany; dippele@klilu.de; 17 Department of Dermatology and Allergy, University Hospital, Ludwig-Maximilians-University (LMU), 80337 Munich, Germany; 18 Novartis Campus Basel, 4056 Basel, Switzerland; manar.aoun@novartis.com; 19 EUMelaReg Consortium, 14057 Berlin, Germany; magdalena.walecki@eumelareg.org; 20 Department of Dermatology, Helios Klinikum Erfurt, 99088 Erfurt, Germany; rudolf.herbst@helios-gesundheit.de; 21 Department of Dermatology, Johannes Wesling Medical Center, Ruhr University Bochum, Campus Minden, 32429 Minden, Germany; yenny.angela@muehlenkreiskliniken.de (Y.A.); rudolf.stadler@muehlenkreiskliniken.de (R.S.); ralf.gutzmer@muehlenkreiskliniken.de (R.G.); 22 Department of Dermatology, University Hospital Regensburg, 93053 Regensburg, Germany; sebastian.haferkamp@klinik.uni-regensburg.de; 23 Department of Dermatology, Skin Cancer Center, General Hospital Karlsruhe, Academic Teaching Hospital of the University of Freiburg, 76187 Karlsruhe, Germany; claus-detlev.klemke@klinikum-karlsruhe.de; 24 Department of Dermatology, Dermatosurgery and Allergology, Klinikum Bremen-Ost, 28195 Bremen, Germany; k.kaune@dermatologikum.de; 25 Department of Dermatology and Venereology, Martin Luther University Halle-Wittenberg, 06120 Halle, Germany; johannes.wohlrab@medizin.uni-halle.de; 26 Department of Dermatology, University Hospital Tuebingen, 72076 Tuebingen, Germany; ulrike.leiter@med.uni-tuebingen.de; 27 Department of Dermatology, Venereology and Allergology, Skin Cancer Center, Ruhr-University Bochum, 44791 Bochum, Germany; nessr.aburached@klinikum-bochum.de; 28 Skin Cancer Unit, German Cancer Research Center (DKFZ), 69120 Heidelberg, Germany; jochen.utikal@umm.de; 29 Department of Dermatology, Venereology and Allergology, University Medical Center Mannheim, Ruprecht-Karl University of Heidelberg, 68167 Mannheim, Germany; 30 DKFZ Hector Cancer Institute, University Medical Center Mannheim, 68167 Mannheim, Germany; 31 Department of Dermatology and Venereology, Helios Klinikum Schwerin, 19055 Schwerin, Germany; gaston.schley@helios-gesundheit.de; 32 Department of Dermatology and Allergy, Harzklinikum Dorothea Christiane Erxleben GmbH, 06484 Quedlinburg, Germany; jens.ulrich@harzklinikum.com; 33 Department of Dermatology and Venereology, Skin Cancer Center, University Medical Center Hamburg-Eppendorf (UKE), 20246 Hamburg, Germany; ch.gebhardt@uke.de; 34 Department of Dermatology, University of Lubeck, 23538 Lubeck, Germany; patrick.terheyden@uksh.de; 35 German Cancer Consortium (DKTK) Partner Site Essen, and National Center for Tumor Diseases (NCT-West) Campus Essen, 45147 Essen, Germany

**Keywords:** adjuvant, adverse event management, dabrafenib, trametinib, melanoma, pyrexia, quality of life

## Abstract

Background/Objectives: Malignant melanoma is a highly aggressive cancer associated with significant mortality, underscoring the need for continued research efforts. COMBI-EU (NCT03944356) is a prospective, non-interventional study that aims to assess adjuvant dabrafenib and trametinib usage in clinical practice, the impact of AE management, and the usage of app-based documentation on treatment adherence. Methods: Adults with complete surgical resection of stage III BRAF V600-mutant cutaneous melanoma were included. The primary endpoint was median time on treatment (TOT). Adverse event (AE) management was classified as either a high or low level of management. The rating of AE management based on a self-developed algorithm and rules from COMBI-APlus was used to analyze the impact of AE management on TOT. App-based documentation of medication intake and patient-reported outcomes (CANKADO PRO-React; version 6.0, 06.03.2019) was offered. Results: For 225 patients, the median TOT was 11.8 months (95% confidence interval [CI]: 11.7, 12.0). Treatment was completed by 138 patients (61.3%); 37 (16.4%) discontinued due to treatment-related AEs (TRAEs). TRAEs (≥1) were experienced by 181 patients (80.4%); the most common was pyrexia (38.2%). High-level AE management showed a trend toward improved treatment adherence (high versus low level: hazard ratio [HR]: 0.74; 95% CI: 0.49, 1.14); this improvement was significant with pyrexia management (HR: 0.52; 95% CI: 0.29, 0.93). Seventy-nine (35%) and 33 patients (15%) intended to use and eventually used the app, respectively. A similar proportion of patients remained on treatment for 12 months irrespective of app usage (use, 39.4% vs. non-use, 36.5%). Conclusions: High-level TRAE management showed a trend toward improved treatment adherence, which was statistically significant for pyrexia. Optional use of an app did not influence treatment adherence.

## Simple Summary

The prospective, non-interventional COMBI-EU study investigated the impact of adverse event management, and app-based health-tracking on the treatment adherence of patients with stage III BRAF V600 mutant cutaneous melanoma. Between July 2019 and December 2023, 225 patients who received adjuvant dabrafenib and trametinib in a real-world clinical practice were included at 31 treatment centers in Germany. High-level treatment-related adverse event management showed a trend toward improved treatment adherence (HR: 0.74; [0.49–1.14]), which was statistically significant for pyrexia. Optional use of a health-tracking app did not affect treatment adherence.:

## 1. Introduction

Melanoma is characterized by high metastatic potential. An estimated 325,000 new cases of cutaneous melanoma were diagnosed in 2020 worldwide [1]. By 2040, new melanoma cases are projected to rise by more than 50%, with deaths increasing by 68% [1].

Surgical excision of the primary tumor and the affected lymph nodes is often the first step in treating patients with AJCC (American Joint Committee on Cancer 8th edition) stage III melanoma [2], but this still leaves a substantial risk of recurrence or progression to distant metastases (stage IV) [3,4,5]. Patients with stage III melanoma show a 5-year melanoma specific survival of less than 40% up to more than 90%, depending on the respective sub-stage [6]. In total, a 5-year overall survival (OS) rate of approximately 55%, and a 5-year distant metastasis-free survival (DMSF) rate of about 40% can be found [7,8]. Therefore, the standard of care for patients with stage III melanoma is to offer adjuvant therapy to reduce the risk of recurrence [3,9]

BRAF mutations are found in approximately 50% of melanoma patients with the most common mutation being BRAF V600E [10,11]. These patients are eligible for treatment with either targeted therapy, which includes BRAF inhibitors (BRAFi) and MEK inhibitors (MEKi), or with immune checkpoint inhibitors (ICI).

Adjuvant treatments either with the BRAF/MEKi combination of dabrafenib and trametinib (D/T) [12,13] or with ICI like nivolumab [14,15] or pembrolizumab [16,17] have shown to prolong recurrence-free survival (RFS) and are standard-of-care options for patients with high-risk resected stage III disease harboring a BRAF V600 mutation [3,9]. Across trials investigating adjuvant therapy, 5-year recurrence-free survival (RFS) rates range from 50% to 55% and 5-year distant metastasis-free survival (DMFS) rates range from 58% to 65% [12,15,16], highlighting the need to better understand the factors influencing treatment success and recurrence. In the phase 3 COMBI-AD study, adjuvant D/T prolonged RFS compared with placebo [12,13,18], with a stable improvement over nearly 10 years of follow-up (hazard ratio [HR] for relapse or death: 0.52; 95% confidence interval [CI]: 0.43, 0.63; HR for distant metastasis or death: 0.56; 95% CI: 0.44, 0.71) [13]. Patients with BRAF V600E mutations experienced the greatest benefit (HR: 0.75; 95% CI: 0.58, 0.96) compared to those with V600K mutations, alongside those with primary tumor ulceration and macro metastasis [13]. Based on these results, the European Society of Medical Oncology Clinical Practice guidelines recommend adjuvant D/T as a standard-of-care adjuvant treatment option for BRAF V600E-mutated stage III melanoma [19]. However, in COMBI-AD, 26% of patients discontinued the 12-month treatment course early due to adverse events (AEs), with pyrexia being the most common reason in 9% of all patients [18]. Treatment adherence may be reduced in the adjuvant setting, where patients generally have no evidence of disease and may therefore have limited tolerance for treatment-related AEs (TRAEs). Factors affecting adherence to treatment can be related to patients, therapies, patients’ socioeconomic and health status, attitudes of healthcare providers, or the healthcare system [20,21]. However, it has been shown that TRAEs are the most relevant factor for treatment adherence in patients receiving adjuvant D/T treatment [18]. Common AEs, such as pyrexia, can limit patients’ ability to receive a full course of adjuvant D/T treatment [18,22] and data from the COMBI-APlus study suggest that better AE management might improve treatment adherence and possibly outcomes [23]. Patient health-tracking apps can improve care by monitoring symptoms, AEs, and health-related quality of life (HRQOL) [24,25,26].

The COMBI-EU (combined use of adjuvant D/T in Europe) study aimed to assess the impact of AE management on treatment adherence in patients treated with adjuvant D/T in a real-world clinical setting, as compared to clinical trials.

## 2. Materials and Methods

### 2.1. Study Design and Patient Selection

**Figure 1:**
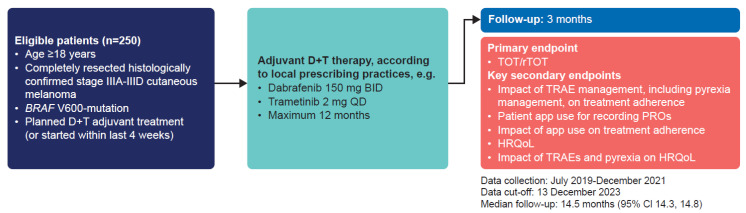
COMBI-EU study design. BID, twice daily; CI, confidence interval; D + T, dabrafenib plus trametinib; HRQoL, health-related quality of life; PRO, patient-reported outcome; QD, once daily; rTOT, TOT censored for recurrence (treatment adherence); TOT, time on treatment; TRAE, treatment-related adverse event.

COMBI-EU (NCT03944356) was a prospective, non-interventional study conducted from July 2019 to December 2023 at 31 treatment centers in Germany, and the data cut-off for this analysis was 13 December 2023 by the EUMelaReg consortium, which is a multinational registry collaboration on the European level aimed at collecting and scientifically evaluating high-quality clinical care data on melanoma and other types of skin cancer. The study design is shown in Figure 1.

Eligible patients aged ≥18 years had completely surgically resected, histologically confirmed AJCC (8th edition) clinical stage IIIA to IIID BRAF V600-mutated cutaneous melanoma, and planned treatment with D/T or had started D/T within 4 weeks of study entry. Patients with current or planned participation in a clinical trial (except participation in a follow-up phase clinical trial without active intervention was permitted) or current or planned treatment for another tumor disease (except keratoacanthoma, squamous cell, or basal cell carcinoma of the skin) were not eligible and therefore excluded.

Treatment with D/T was administered according to local prescribing practices (e.g., dabrafenib 150 mg twice daily and trametinib 2 mg daily) for a maximum of 12 months. Study visits followed the schemes of the respective study site, and the assessments were typically scheduled to take place at the start of treatment, every 4 weeks until week 16 and at months 6, 9, and 12 of treatment, at the end of treatment, and at 3 months after treatment end.

### 2.2. Endpoints and Assessments

The primary endpoints were time on treatment (TOT), defined as the interval between the start of treatment and permanent discontinuation of treatment and recurrence-censored TOT (rTOT), were TOT was censored at treatment stop in a Kaplan–Meier estimation. o reflect treatment adherence, the rTOT over time was used and accordingly the completion rate was defined as the estimated rate of patients completing at least 48 weeks of treatment, when censored for recurrence.

Selected secondary endpoints include the impact of TRAE and pyrexia management level on treatment adherence, the voluntary use of an electronic patient app (CANKADO PRO-React, CANKADO Service GmbH) to record medication intake, current well-being via patient-reported outcomes (PROs), the impact of app use on treatment adherence, and HRQOL, based on the European Organization for Research and Treatment of Cancer Quality of Life Questionnaire (EORTC-QLQ-C30), documented in the CANKADO app for users and on tablets provided by CANKADO during in-person visits for non-users.

The TRAE management level was based on a self-developed algorithm: High: any TRAE managed by dose reduction or attempts to reintroduce at any dose level after interruption. Grade 1 to 2 TRAE managed by symptomatic measures while continuing the current dosage. Low: grade 1 to 2 TRAE or any pyrexia event managed by permanent treatment discontinuation without dose modification or attempts to reintroduce at any dose level after improvement of the TRAE; grade 4 TRAE managed while continuing the current dosage. Not applicable: any other constellations of TRAE and management, e.g., no TRAE, grade 1 TRAE other than pyrexia not affecting treatment delivery, or grade ≥3 TRAE requiring treatment discontinuation.

Pyrexia management level was based on rules from COMBI-APlus [23]. High: pyrexia managed in accordance with COMBI-APlus, e.g., full dose interruption, after which full-dose treatment resumed; Partly: pyrexia managed differently, e.g., by dose reduction of 1 drug only or treatment interruption, after which treatment resumed at a lower dose level; and Low: pyrexia managed by stopping treatment completely or not changing the initial dose at all. Not applicable: no pyrexia.

Efficacy endpoints included RFS, DMFS, and overall survival (OS) times and rates at 12 months. The correlation between TOT and efficacy endpoints was also assessed.

### 2.3. Statistical Analysis

All analyses were performed using the full analysis set, defined as all patients who received ≥1 treatment dose. Sample size was determined based on the estimation of median TOT: assuming 70% of patients would be on treatment at 12 months and a 10% dropout rate, a sample size of 250 patients provides a 95% CI width of 0.12. TOT and other time-to-event parameters (RFS, DMFS, and OS) were calculated by Kaplan–Meier analysis; all other parameters were assessed in an exploratory and descriptive manner. For rTOT, patients who discontinued due to disease recurrence were censored at the last dose received.

Statistical significance between variables was assessed by the chi-squared test, while numerical differences were calculated by the Wilcoxon rank sum test (2 groups) or the Kruskal-Wallis test (>2 groups). Correlations were analyzed using Spearman and linear regression models. No adjustments were made for multiple testing. A type 1 error rate of 0.05 was used.

### 2.4. Ethical Approval and Consent to Participate

The study was performed in accordance with the principles laid down in the Declaration of Helsinki and the standards of the International Conference of Harmonization (ICH) Tripartite Guideline for Good Clinical Practice, Guidelines for Good Epidemiological Practice (GEP), and Good Pharmacoepidemiology Practice. Documented approval from the appropriate independent ethics committee or institutional review board was obtained at participating centers. Written consent was obtained from patients.

## 3. Results

### 3.1. Patient Characteristics

Between July 2019 and December 2023, 242 patients were screened for eligibility for the study. Twelve patients were not deemed eligible: eight patients (66%) did not fulfil the inclusion criteria for clinical stage III melanoma, three patients (25%) did not fulfil the inclusion criteria for adjuvant D/T treatment specification, and one patient (0.8%) did not fulfil the exclusion criteria as he was undergoing treatment for another tumor. Additionally, five patients were excluded after enrollment (two withdrew consent and one died before the start of the study, one refused medication, and one did not participate due to poor left ventricular ejection fraction). A total of 225 patients were analyzed; Table 1 summarizes the baseline characteristics, stratified by stage III category (stage III at primary melanoma diagnosis or as recurrent stage). Of 225 patients, 149 (66%) had a primary stage III diagnosis and 76 (34%) had a recurrent stage III diagnosis. Over half were male (*n* = 129, 57.3%), and the median age of all patients was 58 years (range: 20–87 years). Most patients had an Eastern Cooperative Oncology Group (ECOG) performance status of 0 (n = 205, 91.1%), a BRAF V600E mutation (*n* = 184, 81.8%), nodular melanoma (*n* = 82, 36.4%), or superficial spreading melanoma (*n* = 74, 32.9%).

### 3.2. Treatment Adherence

Treatment adherence was assessed using TOT/rTOT, defined as the time from treatment start to permanent discontinuation for any reason/the first documented disease recurrence. Treatment completion was defined as remaining on therapy for 12 months.

Of 225 patients, 138 (61%) completed at least 12 months of adjuvant D/T. The median time of follow-up was 14.5 months (95% CI: 14.3, 14.8). Most patients (*n* = 151; 67.1%) received >9 months of treatment; only 28 (12.4%) discontinued before 3 months of treatment. The most common reason for discontinuation was TRAEs (*n* = 37, 16.4%; grade 1: 36%, grade 2: 45%, grade 3: 16%, and grade 4: 3%), followed by disease recurrence, (*n* = 25, 11.1%), patient decision, (*n* = 14, 6.2%), physician discretion (*n* = 4, 1.8%), loss to follow-up (*n* = 2, 0.9%), and other reasons (*n* = 5, 2.2%).

**Figure 2:**
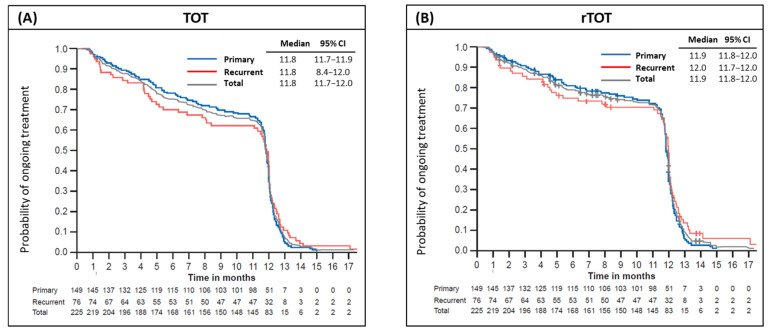
Kaplan–Meier estimates for (**A**) time on treatment (TOT), and (**B**) recurrence TOT (rTOT; time on treatment censored for recurrence) with D/T in patients with primary (blue line) or recurrent (red line) stage III disease at baseline. D/T, dabrafenib and trametinib; CI, confidence interval.

Figure 2A,B illustrate TOT and rTOT, highlighting trends in treatment adherence over time. The median TOT was 11.8 months (95% CI: 11.7, 12.0); after censoring for recurrence, the median rTOT was 11.9 months (95% CI: 11.8, 12.0) with no significant difference observed between the primary and recurrent groups. The estimated overall completion rate for treatment at 12 months with censoring for recurrences was 72%.

### 3.3. Treatment Adherence Assessed by TRAE Management

**Figure 3:**
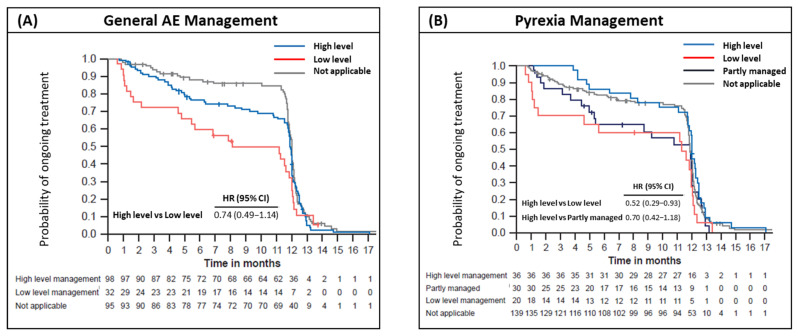
Kaplan–Meier estimates for real-world time on treatment censored for recurrence (rTOT) by (**A**) AE management, and (**B**) pyrexia management for high-level management (blue line), low-level management (red line), partly managed (dark blue line), and not applicable (grey line). High-level management of AE and pyrexia was associated with improved treatment adherence compared with low-level management. AE, adverse event; HR, hazard ratio; CI, confidence interval.

The occurrence of TRAEs is shown in Table 2. TRAEs occurred in 181 patients (80.4%). The most common TRAEs (>25%, grade 1–2) were increased liver enzymes (*n* = 91, 40.4%), fever (*n* = 68, 30.2%), and fatigue (*n* = 61, 27.1%). A total of 98 patients (43.6%) received high-level TRAE management, and 32 (14.2%) had low-level management; all remaining patients were included in the “Not applicable” group, which included no TRAE, grade 1 TRAE not affecting treatment delivery, or grade ≥3 TRAE requiring treatment discontinuation. High-level TRAE management was associated with improved treatment adherence compared with low-level AE management (HR: 0.74; 95% CI: 0.49, 1.14; Figure 3A). Among patients with relevant and manageable TRAEs, the completion rate for treatment was 69% with high-level management and 49% with low-level management, respectively.

### 3.4. Treatment Adherence Assessed by Pyrexia Management

Occurrence of at least one pyrexia event was reported in 86 patients (38.2%), with grade 1 (57.0%), grade 2 (39.5%), and grade ≥3 (2.3%). The median time to first occurrence of pyrexia was 17 days (range: 1–279 days), and the median duration of first occurrence of pyrexia was 4 days (range: 1–82 days). In 70% of pyrexia cases, the event occurred in the first 30 days of treatment (Supplementary Figure S1). Thirty-six patients (16.0%) received high-level pyrexia management, 30 (13.3%) were partly managed, and 20 (8.9%) received low-level management; all remaining patients were included in the “Not applicable” group. High-level pyrexia management was associated with significantly improved treatment adherence compared with low-level pyrexia management (HR: 0.52; 95% CI: 0.29, 0.93; Figure 3B). Completion rates for treatment among patients receiving high-level, low-level, and partial management were 75%, 60%, and 57%, respectively.

### 3.5. App Use

**Figure 4:**
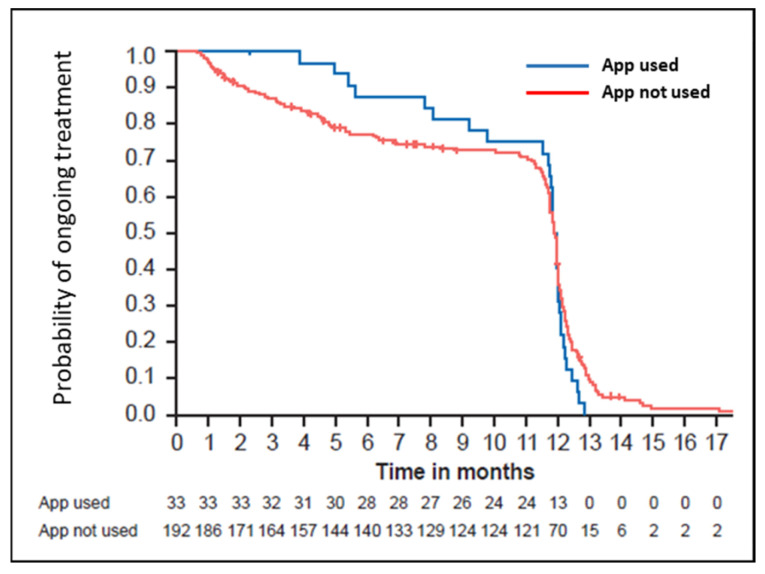
Kaplan–Meier estimates for real-world time on treatment by health-tracking app users (blue line) and non-users (red line).

We used the CANKADO app to address treatment adherence. Patients included in the study could voluntarily use the provided app, a digital health platform and patient support tool designed to help users document their medication intake and physical well-being. It supports symptom tracking, documentation of PROs, and therapy adherence. Of the 225 patients, 79 patients (35%) indicated that they intended to use the app during the study; however, only 33 (15%) used the app. Baseline characteristics were similar between users and non-users (Supplementary Table S1), except that app users were significantly younger than non-users (median age: 55 years [range: 20–75 years] vs. 59 years [range: 24–87 years]). The completion rate for treatment was 75% in app users and 71% in non-users (Figure 4). A similar proportion of app users and non-users received high-level TRAE management (51.5% vs. 42.2%; *p* = 0.52). Pyrexia was reported in 16 of 33 app users (48.5%) and 70 of 192 non-users (36.5%). Use of high-level pyrexia management was similar in users and non-users (18.2% vs. 15.6%; *p* = 0.462).

### 3.6. Health-Related Quality of Life

**Figure 5:**
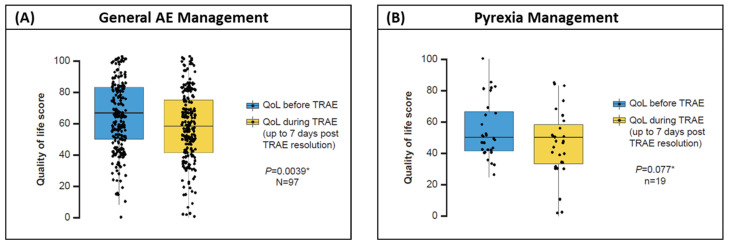
Effect of experiencing a (**A**) TRAE or (**B**) pyrexia on HRQoL scores (EORTC-QLQ-C30). Each data point represents one TRAE/pyrexia/recurrence episode. * Wilcoxon matched pairs test. N, number of patients; EORTC-QLQ-C30, European Organization for Research and Treatment of Cancer Quality of Life Questionnaire; HRQoL, health-related quality of life; QoL, quality of life; TRAE, treatment-related adverse event.

Changes in EORTC-QLQ-C30 baseline functioning and symptom sub scores remained stable throughout the 12-month treatment period (Supplementary Figure S2); the low incidence of grade 3 to 4 TRAEs and the lack of long-term AEs may have had a positive effect on HRQOL. Experiencing a TRAE was associated with significant worsening in the EORTC-QLQ-C30 summary score (*p* = 0.0039; Figure 5A). There was no significant association between pyrexia and HRQOL (*p* = 0.077; Figure 5B).

### 3.7. Efficacy Outcomes

Kaplan–Meier estimates of RFS, DMFS, and OS for patients with primary and recurrent disease are shown in Supplementary Figure S3. Median RFS, DMFS, and OS were not reached at the time of the analysis. The analysis of treatment adherence and efficacy outcomes could not be performed due to the short 3-month follow-up period.

## 4. Discussion

Our results indicated that high-level AE management, particularly for pyrexia, was associated with improved adherence to adjuvant D/T for resected stage III BRAF V600-mutant melanoma. The results also showed a high completion rate (72%) of adjuvant D/T in clinical practice. The discontinuation rates due to AEs for D/T were 15.4% and 16.1%, respectively, which compare favorably with the 26% rate observed in COMBI-AD [18]. A similarly low rate of discontinuation due to AEs (16%) was seen in the COMBI-APlus study evaluating a pyrexia management algorithm [23]. Furthermore, COMBI-APlus demonstrated improvement in the incidence of composite pyrexia events compared with COMBI-AD; 44 patients (8.0%) experienced grade 3 or 4 pyrexia, hospitalization, or permanent discontinuation due to pyrexia compared with 20.0% in COMBI-AD [23]. In a retrospective registry study of 65 patients receiving adjuvant D/T at 25 hospitals in Spain (DESCRIBE-AD), only 6 patients (9.2%) discontinued adjuvant D/T due to AEs [27].

In this study, 80% of patients experienced TRAEs, of which most events were mild or moderate in severity, compared with 92% and 93% experiencing TRAEs in COMBI-AD and COMBI-APlus, respectively [13,23]. The lower rate of TRAEs might well be explained by the less rigorous uptake of AEs in an observational study compared to a clinical trial. High-level TRAE management was associated with improved treatment adherence compared with low-level management, indicating that effective measures to control TRAEs can allow more patients to complete the full course of treatment. Notably, high-level pyrexia management, using rules from COMBI-APlus [23,28], was associated with improved treatment adherence compared with low-level management. In the COMBI-APlus study, the pyrexia intervention strategy involved prompt interruption of D/T therapy when pyrexia syndrome occurred (≥38 °C) and restarting it at the same dose once patients had been symptom-free for at least 24 h and their temperature was below 38 °C. In addition, supportive care included antipyretics and corticosteroids (if the fever did not resolve with antipyretics alone). Pyrexia is well documented as the most common and mostly treatment-related AE observed with D/T [18,29], and better management of pyrexia could help patients to remain on treatment longer. In a 4-year follow-up of a multicenter study that evaluated the disease and treatment course of patients with stage III melanoma who received either programmed cell death protein-1 (PD1) inhibition or targeted therapy with BRAF/MEK inhibitors, those who discontinued adjuvant targeted therapy within 6 months (except for progressive disease) had a higher risk of rapid recurrence compared with those who continued treatment [30], highlighting the importance of treatment adherence.

Regarding the electronic app, there was only a limited number of patients who expressed interest in using the app, and only 15% used it during the study. Therefore, app use in this patient population had a minimal impact on treatment adherence, although the number of users was small and selection bias cannot be ruled out, since younger patients were more likely to use the app than older patients. This highlights challenges in using digital support tools in real-world settings, especially among older populations. Several studies have shown mixed outcomes regarding the impact of electronic PRO platforms in cancer care [24,26,31,32,33,34].

The results from this study suggest that in a real-world setting, adjuvant D/T had a limited impact on HRQOL. Experiencing TRAEs was associated with a modest worsening of HRQOL, emphasizing the importance of TRAE management. Similarly, in COMBI-AD, adjuvant D/T did not have a relevant effect on PROs during the treatment or long-term follow-up (range: 15–48 months) [35] between patients who did and did not experience AEs. However, in COMBI-AD, recurrence was associated with worsening of EQ-5D-3L VAS and utility scores in both treatment arms [18]. In this study, the lack of impact of recurrence on HRQOL was unexpected and requires further evaluation. In COMBI-APlus, no clear worsening of HRQOL was observed during the treatment, except at relapse [36]. Overall, the HRQOL findings support adjuvant D/T in this setting.

Data on RFS and OS in those with primary disease from COMBI-EU are consistent with findings from COMBI-AD [18], where the 1-year RFS rate was 88%, and the 1-year OS rate was 97%. Similarly, high 1-year RFS and OS rates of 95.3% and 100%, respectively, were reported from DESCRIBE-AD [27].

This was a non-interventional, observational study, without a control group; therefore, the relationship between the results and the effects of exposure to adjuvant D/T cannot be confirmed. The optional use of the patient app does not allow for conclusions to be drawn regarding its correlation with treatment adherence.

A limitation of this study was that it was conducted in a single country, which may limit the generalizability of the findings to melanoma populations managed under different healthcare systems. National differences in D/T experience can affect AR management, and access to adjuvant systemic therapies may have influenced treatment selection, recurrence detection, and outcomes. Therefore, these results should be interpreted within the context of country-specific melanoma care pathways. Another potential bias could arise from the inclusion of patients who had already started D/T therapy up to 4 weeks earlier, potentially underrepresenting patients with very early treatment stop. However, patients with less than 4 weeks of treatment were still eligible for inclusion, thereby mitigating this kind of bias. Also, the association of side effect management with treatment is merely associative and does not prove causality.

## 5. Conclusions

Adherence was relatively high in this real-world study of adjuvant D/T in resected BRAF V600-mutant melanoma. High-level TRAE management, particularly for pyrexia, was associated with improved treatment adherence. Only a small subgroup of patients opted for the use of an app that did not appear to influence treatment adherence.

## 6. Future Directions

Currently, it is unknown whether the completion of one year of treatment with adjuvant D/T is required for achieving efficacy, or whether there is a continuous relationship between treatment duration and efficacy. Further research, including long-term outcomes of patients with optimized treatment adherence, is needed to understand the impact of treatment adherence on the effectiveness of adjuvant D/T.

## Figures and Tables

**Table 1:** Patient baseline characteristics.

Baseline Characteristics	Primary (N = 149)	Recurrent (N = 76)	*p*-Value	Overall (N = 225)
Sex			0.76	
Female	62 (41.6%)	34 (44.7%)		96 (42.7%)
Male	87 (58.4%)	42 (55.3%)		129 (57.3%)
Age (years)			0.07	
Median (Min; Max)	57 (24; 83)	60.5 (20; 87)		58 (20; 87)
ECOG			0.39	
0	133 (89.3%)	72 (94.7%)		205 (91.1%)
1	12 (8.1%)	3 (3.9%)		15 (6.7%)
≥2	4 (2.7%)	1 (1.3%)		5 (2.2%)
Melanoma subtype			0.04	
SSM	45 (30.2%)	29 (38.2%)		74 (32.9%)
NMM	61 (40.9%)	21 (27.6%)		82 (36.4%)
MUP	11 (7.4%)	1 (1.3%)		12 (5.3%)
ALM	3 (2.0%)	6 (7.9%)		9 (4.0%)
UCM	2 (1.3%)	3 (3.9%)		5 (2.2%)
Cutaneous, unspecified	19 (12.8%)	12 (15.8%)		31 (13.8%)
Cutaneous, other subtype	8 (5.4%)	4 (5.3%)		12 (5.3%)
Ulceration of primary tumor			0.02	
Yes	69 (46.3%)	31 (40.8%)		100 (44.4%)
No	69 (46.3%)	41 (53.9%)		110 (48.9%)
Unknown	-	3 (3.9%)		3 (1.3%)
Not applicable *	11 (7.4%)	1 (1.3%)		12 (5.3%)
AJCC stage 8th edition			<0.001	
Stage IIIA	28 (18.8%)	-		28 (12.4%)
Stage IIIB	46 (30.9%)	25 (32.9%)		71 (31.6%)
Stage IIIC	70 (47.0%)	48 (63.2%)		118 (52.4%)
Stage IIID	5 (3.4%)	3 (3.9%)		8 (3.6%)
Type of LN involvement ^†^			<0.001	
Microscopic ^†^	112 (75.2%)	-		112 (49.8%)
Macroscopic ^‡^	28 (18.8%)	43 (56.6%)		71 (31.6%)
Number of LN involved			0.22	
1	100 (67.1%)	24 (31.6%)		124 (55.1%)
2	25 (16.8%)	9 (11.8%)		34 (15.1%)
3	3 (2.0%)	2 (2.6%)		5 (2.2%)
≥4	10 (6.7%)	7 (9.2%)		17 (7.6%)
Unknown	3 (2.0%)	2 (2.6%)		5 (2.2%)
Size of the largest sentinel LN			<0.001	
<1 mm	31 (20.8%)	-		31 (13.8%)
≥1 mm	70 (47.0%)	-		70 (31.1%)
Unknown	14 (9.4%)	-		14 (6.2%)
In-transit disease			<0.001	
Yes	9 (6.0%)	32 (42.1%)		41 (18.2%)
Yes, microscopic ^†^	4 (2.7%)	-		4 (1.8%)
Yes, macroscopic ^‡^	2 (1.3%)	11 (14.5%)		13 (5.8%)
No	134 (89.9%)	33 (43.4%)		167 (74.2%)
BRAF mutation			0.66	
V600E	126 (84.6%)	58 (76.3%)		184 (81.8%)
V600K	12 (8.1%)	10 (13.2%)		22 (9.8%)
V600D	2 (1.3%)	1 (1.3%)		3 (1.3%)
V600R	2 (1.3%)	1 (1.3%)		3 (1.3%)
Other variants	6 (4.0%)	5 (6.6%)		11 (4.9%)

* Patients with MUP. ^†^ Microscopic is defined as a lymph node metastasis measuring 0.2–2 mm in size. ^‡^ Macroscopic is defined as a lymph node metastasis measuring >2 mm in size. N, number of patients; Min, minimum; Max, maximum; ECOG, Eastern Cooperative Oncology Group; SSM, superficial spreading melanoma; NMM, nodular malignant melanoma; MUP, melanoma of unknown primary; ALM, acral lentiginous melanoma; UCM, unclassifiable melanoma; AJCC, American Joint Committee on Cancer; LN, lymph node; BRAF, BRAF mutation status.

**Table 2:** TRAEs occurring in >5% of patients (any grade) or in ≥1 patient (grade 3–4).

	N = 225		
Adverse Event	Grade 1–2	Grade 3–4	All Grades
Patients with ≥1 TRAEs	181 (80.4%)	NR	181 (80.4%)
General disorders			
Fever	68 (30.2%)	3 (1.3%)	68 (30.2%)
Chills	47 (20.9%)	-	47 (20.9%)
Fatigue	61 (27.1%)	-	61 (27.1%)
General disorders—other	13 (5.8%)	3 (1.3%)	16 (7.1%)
Gastrointestinal disorders			
Nausea	43 (19.1%)	-	43 (19.1%)
Vomiting	13 (5.8%)	1 (0.4%)	14 (6.2%)
Diarrhea	27 (12.0%)	2 (0.9%)	28 (12.4%)
Investigations			
Liver enzymes increased	82 (36.4%)	11 (4.9%)	91 (40.4%)
Creatine phosphokinase increased	49 (21.8%)	6 (2.7%)	53 (23.6%)
Musculoskeletal disorders			
Muscle cramp	14 (6.2%)	-	14 (6.2%)
Nervous system disorders			
Headache	29 (12.9%)	1 (0.4%)	30 (13.3%)
Skin disorders	28 (12.4%)	-	28 (12.4%)
Eye disorders			
Retinal detachment	-	2 (0.9%)	2 (0.9%)
Vascular disorders			
Hypertension	1 (0.4%)	5 (2.2%)	5 (2.2%)

Occurrence of common adverse drug reactions (grade 1 or 2, >5% of patients) or severe adverse drug reactions (grade 3 or 4, in more than one patient) grouped by grade of adverse drug reaction (aggregated into grade 1–2 and grade 3–4). Subcategories may overlap. N, number of patients; TRAE, treatment-related adverse event; NR, not reported.

## Data Availability

Data are contained within the article and Supplementary Materials.

## Supplementary Materials

The following supporting information can be downloaded at: https://www.mdpi.com/article/10.3390/cancers18040667/s1. Figure S1: Cumulative proportion of patients with (A) pyrexia events over time and (B) total number of pyrexia events per month after start of treatment. Data are for 86 patients with ≥1 pyrexia event; Figure S2: Change from baseline in (A) EORTC-QLQ-C30 functioning scores and (B) symptom scores during treatment; Figure S3: Kaplan–Meier estimates of (A) RFS, (B) DMFS, and (C) OS according to disease status at study entry (primary or recurrent); Table S1: Patient baseline characteristics according to app usage.

## Abbreviations

The following abbreviations are used in this manuscript:

AE	Adverse event
AJCC	American Joint Committee on Cancer
CI	Confidence interval
DMFS	Distant metastasis-free survival
D/T	Dabrafenib and trametinib
ECOG	Eastern Cooperative Oncology Group
EORTC-QLQ	European Organization for Research and Treatment of Cancer Quality of Life Questionnaire
EUMelaReg	European Melanoma Registry
GEP	Guidelines for Good Epidemiological Practice
HRQOL	Health-related quality of life
HR	Hazard ratio
ICH	International Conference of Harmonization
LN	Lymph node
Max	Maximum
Min	Minimum
MUP	Melanoma of unknown primary
NMM	Nodular malignant melanoma
NR	Not reported
OS	Overall survival
PRO	Patient reported outcome
PD1	Programmed cell death protein-1
RFS	Recurrence-free survival
rTOT	Recurrence time on treatment
SSM	Superficial spreading melanoma
TRAE	Treatment-related adverse event
TOT	Time on treatment
UCM	Unclassifiable melanoma
