# Supplementary material for: COMBI-EU: Real-World Evidence on Adverse Event Management and Time on Therapy with Adjuvant Dabrafenib Plus Trametinib in Patients with BRAF V600-Mutant Melanoma
Source: Cancers (Basel). 2026 Feb 18;18(4):667. doi: 10.3390/cancers18040667 (PMC12939694; doi:10.3390/cancers18040667)
Supplement: Supplementary file 1 [file cancers-18-00667-s001.zip › cancers-4122771-supplementary.pdf]

Supplementary Appendix

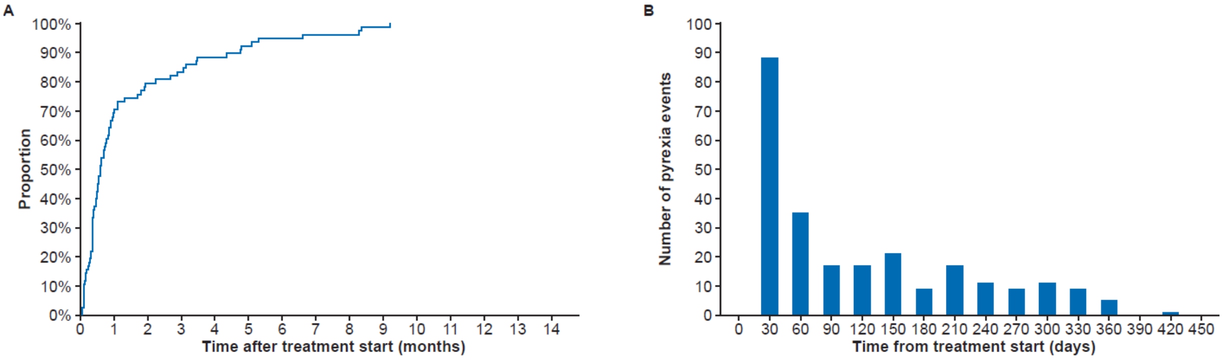

Supplementary Figure S1. Cumulative proportion of patients with (A) pyrexia events over time and (B) total number of pyrexia events per month after start of treatment. Data are for 86 patients with ≥1 pyrexia event.

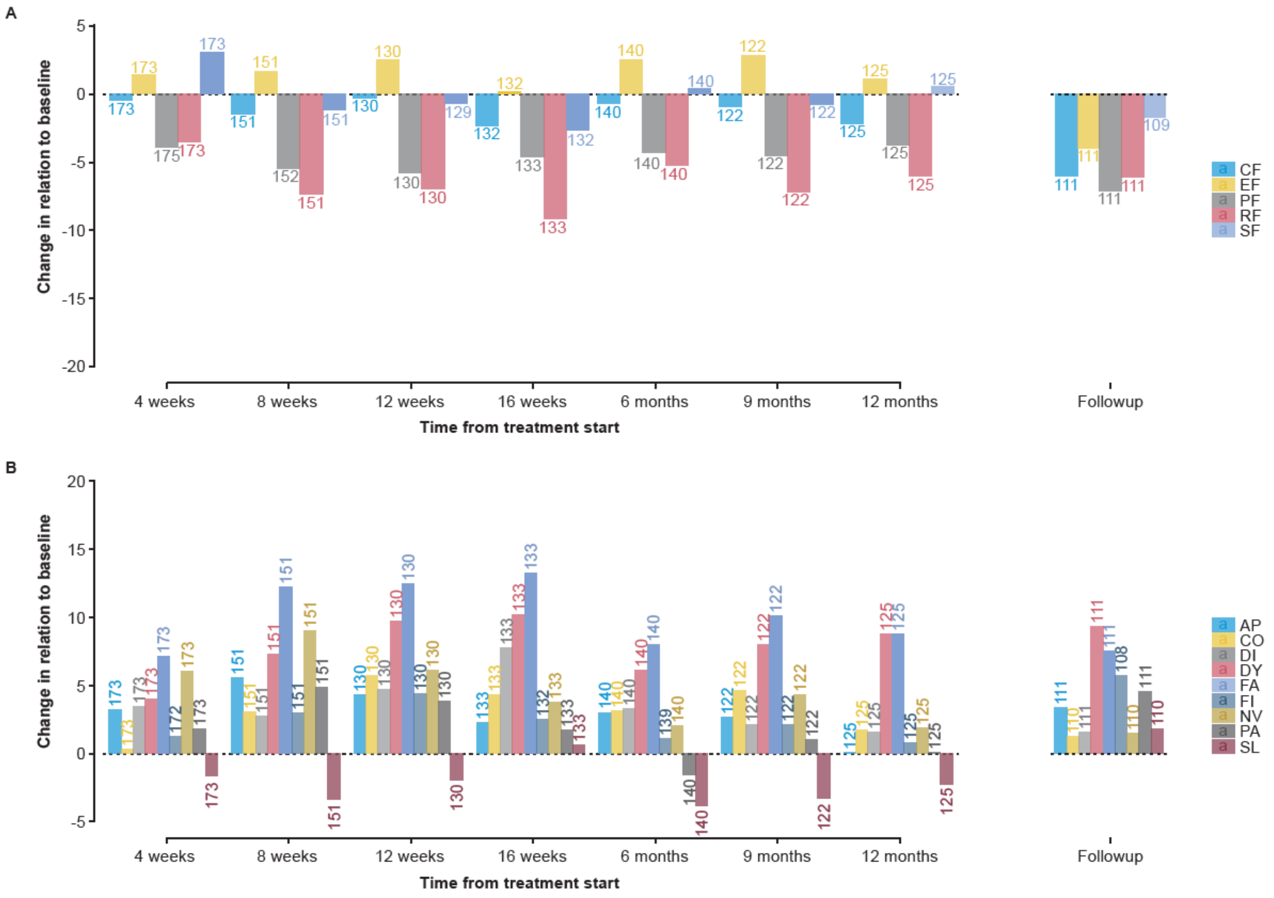

Supplementary Figure S2. Change from baseline in (A) EORTC-QLQ-C30 functioning scores and (B) symptom scores during treatment. The first missing value in a series of missing values was imputed by a last observation carried forward (LOCF) algorithm. The follow-up visit includes data from the last questionnaire received after treatment end (+10 days). Numbers on bars indicate the number of patients analyzed. AP, appetite loss; CF, cognitive functioning; CO, constipation; DI, diarrhea; DY, dyspnea; EF, emotional functioning; EORTC-QLQ-C30, European Organization for Research and Treatment of Cancer Quality of Life Questionnaire; FA, fatigue; FI, financial difficulties; NV, nausea and vomiting; PA, pain; PF, physical functioning; RF, role functioning; SF, social functioning; SL, insomnia.

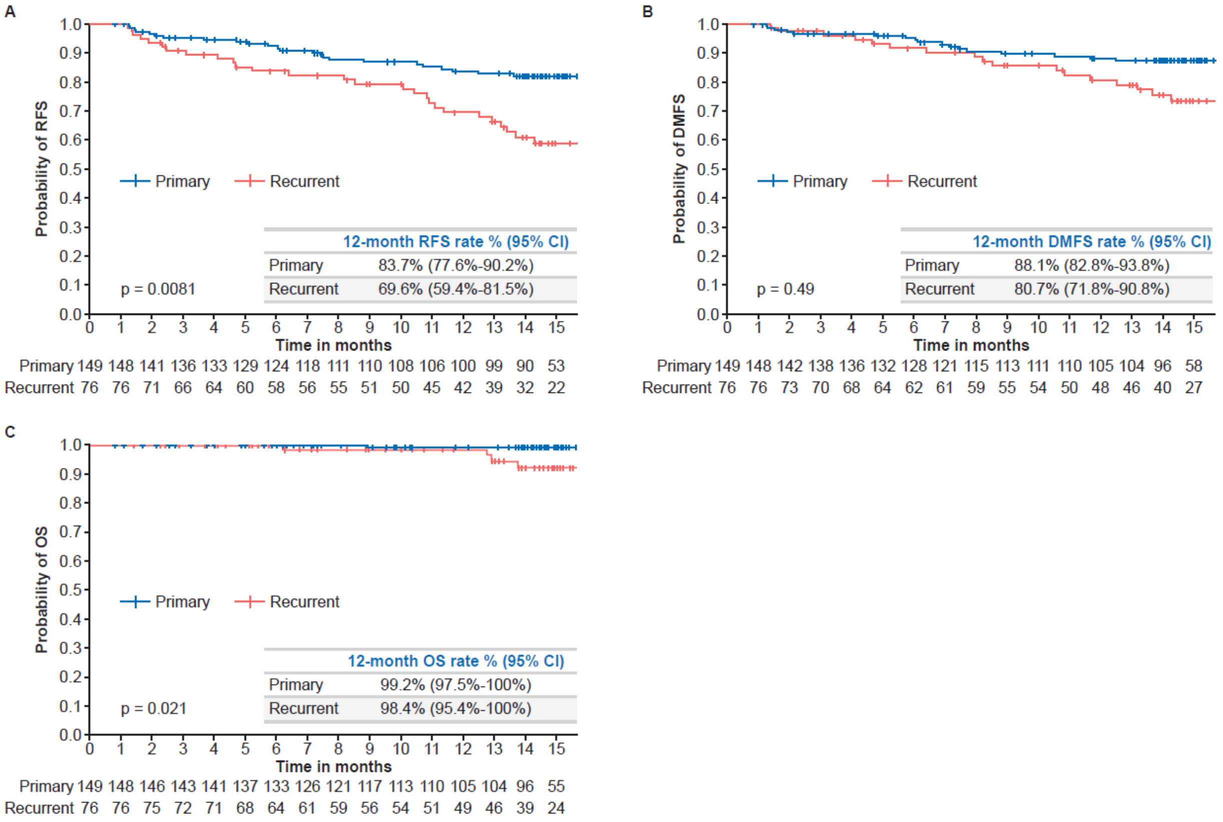

Supplementary Figure S3. Kaplan–Meier estimates of (A) RFS, (B) DMFS, and (C) OS according to disease status at study entry (primary or recurrent). CI, confidence interval; DMFS, distant metastasis-free survival; RFS, recurrence-free survival; OS, overall survival.

Supplementary Table S1. Patient baseline characteristics according to app usage.

Characteristics	App user (N = 33)	App non-user (N = 192)	P-value
**Sex**			
Female	12 (36.4%)	84 (43.8%)	0.55
Male	21 (63.6%)	108 (56.3%)	
**Age (years)**			
Median (Min; Max)	55 (20; 75)	59 (24; 87)	0.03
**ECOG**			
0	30 (90.9%)	175 (91.1%)	0.55
1	3 (9.1%)	12 (6.3%)	
≥2	-	5 (2.6%)	
**Melanoma subtype**			
MUP	4 (12.1%)	8 (4.2%)	
SSM	9 (27.3%)	65 (33.9%)	0.62
NMM	11 (33.3%)	71 (37.0%)	
ALM	2 (6.1%)	7 (3.6%)	
UCM	1 (3.0%)	4 (2.1%)	
Cutaneous, unspecified	4 (12.1%)	27 (14.1%)	
Cutaneous, other subtype	2 (6.1%)	10 (5.2%)	
**Ulceration of primary tumor**			
Yes	14 (42.4%)	86 (44.8%)	0.26
No	15 (45.5%)	95 (49.5%)	
Unknown	-	3 (1.6%)	
Not applicable*	4 (12.1%)	8 (4.2%)	
**AJCC stage 8th edition**			
Stage IIIA	4 (12.1%)	24 (12.5%)	0.21
Stage IIIB	15 (45.5%)	56 (29.2%)	
Stage IIIC	14 (42.4%)	104 (54.2%)	
Stage IIID	-	8 (4.2%)	
**Type of LN involvement**			
Microscopic	19 (57.6%)	93 (48.4%)	0.40
Macroscopic	8 (24.2%)	63 (32.8%)	
**Number of LN involved**			
1	23 (69.7%)	101 (52.6%)	0.17
2	4 (12.1%)	30 (15.6%)	
3	-	5 (2.6%)	
≥4	-	17 (8.9%)	
Unknown	-	5 (2.6%)	
**In-transit disease**			
Yes	6 (18.2%)	35 (18.2%)	0.32
Yes + microscopic	-	4 (2.1%)	
Yes + macroscopic	4 (12.1%)	9 (4.7%)	
No	23 (69.7%)	144 (75.0%)	
**BRAF mutation**			
V600E	24 (72.7%)	160 (83.3%)	0.66
V600K	4 (12.1%)	18 (9.4%)	

V600D	-	3 (1.6%)
V600R	1 (3.0%)	2 (1.0%)
Other variants	3 (9.1%)	8 (4.2%)

*Patients with MUP. N, number of patients; SD, standard deviation; ECOG, Eastern Cooperative Oncology Group; MUP, melanoma of unknown primary; SSM, superficial spreading melanoma; NMM, nodular malignant melanoma; ALM, acral lentiginous melanoma; UCM, unclassifiable melanoma; AJCC, American Joint Committee on Cancer; LN, lymph node; BRAF, BRAF mutation status.
